# Cas12a/Guide RNA-Based Platforms for Rapidly and Accurately Identifying Staphylococcus aureus and Methicillin-Resistant S. aureus

**DOI:** 10.1128/spectrum.04870-22

**Published:** 2023-03-21

**Authors:** Xiaoying Cao, Yanbin Chang, Chunqing Tao, Sen Chen, Qiuxia Lin, Chao Ling, Shifeng Huang, Hengshu Zhang

**Affiliations:** a Department of Plastic and Burn Surgery, the First Affiliated Hospital of Chongqing Medical University, Chongqing, People’s Republic of China; b Department of Clinical Laboratory, Gansu Provincial Hospital, Lanzhou, People’s Republic of China; c Department of Clinical Laboratory, the First Affiliated Hospital of Chongqing Medical University, Chongqing, People’s Republic of China; Texas A&M University

**Keywords:** *Staphylococcus aureus*, methicillin-resistant *Staphylococcus aureus*, CRISPR-Cas12a, isothermal amplification, polymerase chain reaction, loop-mediated isothermal amplification, recombinase polymerase amplification, lateral-flow strips

## Abstract

In order to ensure the prevention and control of methicillin-resistant Staphylococcus aureus (MRSA) infection, rapid and accurate detection of pathogens and their resistance phenotypes is a must. Therefore, this study aimed to develop a fast and precise nucleic acid detection platform for identifying S. aureus and MRSA. We initially constructed a CRISPR-Cas12a detection system by designing single guide RNAs (sgRNAs) specifically targeting the thermonuclease (*nuc*) and *mecA* genes. To increase the sensitivity of the CRISPR-Cas12a system, we incorporated PCR, loop-mediated isothermal amplification (LAMP), and recombinase polymerase amplification (RPA). Subsequently, we compared the sensitivity and specificity of the three amplification methods paired with the CRISPR-Cas12a system. Finally, the clinical performance of the methods was tested by analyzing the fluorescence readout of 111 clinical isolates. In order to visualize the results, lateral-flow test strip technology, which enables point-of-care testing, was also utilized. After comparing the sensitivity and specificity of three different methods, we determined that the *nuc*-LAMP-Cas12a and *mecA*-LAMP-Cas12a methods were the optimal detection methods. The *nuc*-LAMP-Cas12a platform showed a limit of detection (LOD) of 10 aM (~6 copies μL^−1^), while the *mecA*-LAMP-Cas12a platform demonstrated a LOD of 1 aM (~1 copy μL^−1^). The LOD of both platforms reached 4 × 10^3^ fg/μL of genomic DNA. Critical evaluation of their efficiencies on 111 clinical bacterial isolates showed that they were 100% specific and 100% sensitive with both the fluorescence readout and the lateral-flow readout. Total detection time for the present assay was approximately 80 min (based on fluorescence readout) or 85 min (based on strip readout). These results indicated that the *nuc*-LAMP-Cas12a and *mecA*-LAMP-Cas12a platforms are promising tools for the rapid and accurate identification of S. aureus and MRSA.

**IMPORTANCE** The spread of methicillin-resistant Staphylococcus aureus (MRSA) poses a major threat to global health. Isothermal amplification combined with the *trans*-cleavage activity of Cas12a has been exploited to generate diagnostic platforms for pathogen detection. Here, we describe the design and clinical evaluation of two highly sensitive and specific platforms, *nuc*-LAMP-Cas12a and *mecA*-LAMP-Cas12a, for the detection of S. aureus and MRSA in 111 clinical bacterial isolates. With a limit of detection (LOD) of 4 × 10^3^ fg/μL of genomic DNA and a turnaround time of 80 to 85 min, the present assay was 100% specific and 100% sensitive using either fluorescence or the lateral-flow readout. The present assay promises clinical application for rapid and accurate identification of S. aureus and MRSA in limited-resource settings or at the point of care. Beyond S. aureus and MRSA, similar CRISPR diagnostic platforms will find widespread use in the detection of various infectious diseases, malignancies, pharmacogenetics, food contamination, and gene mutations.

## INTRODUCTION

Staphylococcus aureus, a Gram-positive facultative anaerobic coccoid bacterium, is a commensal bacterial pathogen widely populating the skin, skin glands, and mucous membranes of healthy individuals, particularly in the nostrils and stomachs (1). In certain instances, nonpathogenic (or commensal) S. aureus can switch from a commensal stage to a pathogenic state. As a prominent human pathogen on a global scale, S. aureus is responsible for a variety of skin and soft tissue infections, as well as respiratory infections, and can cause life-threatening bacteremia, endocarditis, and osteomyelitis (2–4). Mortality and morbidity associated with S. aureus infections decreased significantly after the introduction of penicillin during the 1940s (5). However, the use and overprescribing of penicillin contributed to the emergence and spread of penicillin-resistant S. aureus due to β-lactamase production (6). Decades after its initial description, methicillin-resistant S. aureus (MRSA) emerged as a major cause of hospital-acquired infections worldwide (7). Presently, there is a progressive transition from nosocomial to community-acquired MRSA infections (8). Furthermore, the prevalence of community-acquired MRSA infections has steadily increased, posing significant public health challenges (9, 10). Infections caused by MRSA both in health care settings and in the community result in severe morbidity and high mortality. As reported by 44% of WHO member states (85 in total), the prevalence of MRSA varies from 20% to 80% in some of these countries (11). Likewise, it has been observed that MRSA infections increase patient mortality by 64% compared to infections caused by nonresistant strains (12). Consequently, the high burden of morbidity and mortality has contributed to the overprescription of vancomycin, the last-resort treatment for MRSA infections (10, 13). Inappropriate use of vancomycin is also responsible for the emergence of vancomycin-intermediate S. aureus (VISA) and vancomycin-resistant S. aureus (VRSA) strains (14–16). Due to the rapid development of antibiotic resistance and the lack of therapeutic options, the treatment of MRSA infections has become extremely challenging. Consequently, the appropriate use of antibiotics is a crucial measure to reduce the development and spread of resistance. To guarantee the optimal administration of antibiotics, accurate detection and identification of pathogens and their resistance phenotypes are deemed essential.

Standard approaches for MRSA detection include phenotypic and molecular tests (17, 18). Traditional phenotypic detection methods consist of the cefoxitin disk screening test, the chromogenic agar screening test, and the enrichment broth screening test (i.e., BacLite rapid MRSA test) (19). Recently, a bacteriophage-based assay that detects the amplification of S. aureus-specific bacteriophages in the presence of methicillin was identified as a promising new phenotypic detection approach (20). Despite meeting the criteria for MRSA detection standards, this culture-based method has significant disadvantages, including time consumption, huge volumes of medical waste, and low sensitivity due to the absence of typical bacterial colonies (21–23). Several DNA-based molecular methods have been employed for MRSA detection, including PCR, real-time PCR, multiplex PCR, gene-probe hybridization (e.g., the EVIGENE MRSA kit), loop-mediated isothermal amplification (LAMP), recombinase polymerase amplification (RPA), and the microfluidic digital RPA Slip Chip. For MRSA detection, these sequence-specific detection methods often employ target genes such as the *SCCmec*-*orfX* junction and the *mecA* and staphylococcal protein A (*spa*) genes (10). Recently, matrix-assisted laser desorption–ionization time-of-flight mass spectrometry (MALDI-TOF MS) has been exploited to detect MRSA (24). Whole-genome sequencing (WGS) technology is a groundbreaking tool in clinical and public health microbiology and has been widely used to identify bacterial species and investigate bacterial antibiotic resistance. Although these molecular detection methods are sensitive and specific, the majority need expensive reagents, specialized staff, and complex equipment, rendering them unsuitable for deployment in limited-resource settings.

This study intended to develop a rapid and accurate MRSA detection method suited for use in limited-resource regions. Recently, the system using clustered regularly interspaced short palindromic repeats (CRISPR) and CRISPR-associated proteins (Cas) for nucleic acid detection was created. Among the Cas proteins, Cas12a is able to cleave single-stranded DNA probes for nucleic acid detection when its nonspecific RNA-guided DNase activity is activated by specific *cis* target hybridization with a single guide RNA (sgRNA)-programmed target gene (25). In addition, when paired with nucleic acid amplification techniques, the CRISPR-Cas12a system could detect DNA targets with single-base resolution and high sensitivity (26). Using the CRISPR-Cas12a system in combination with the PCR, LAMP, and RPA techniques, we have developed two rapid and accurate nucleic acid detection platforms for S. aureus and MRSA.

## RESULTS

### Schematic overview of the study.

**FIG 1:**
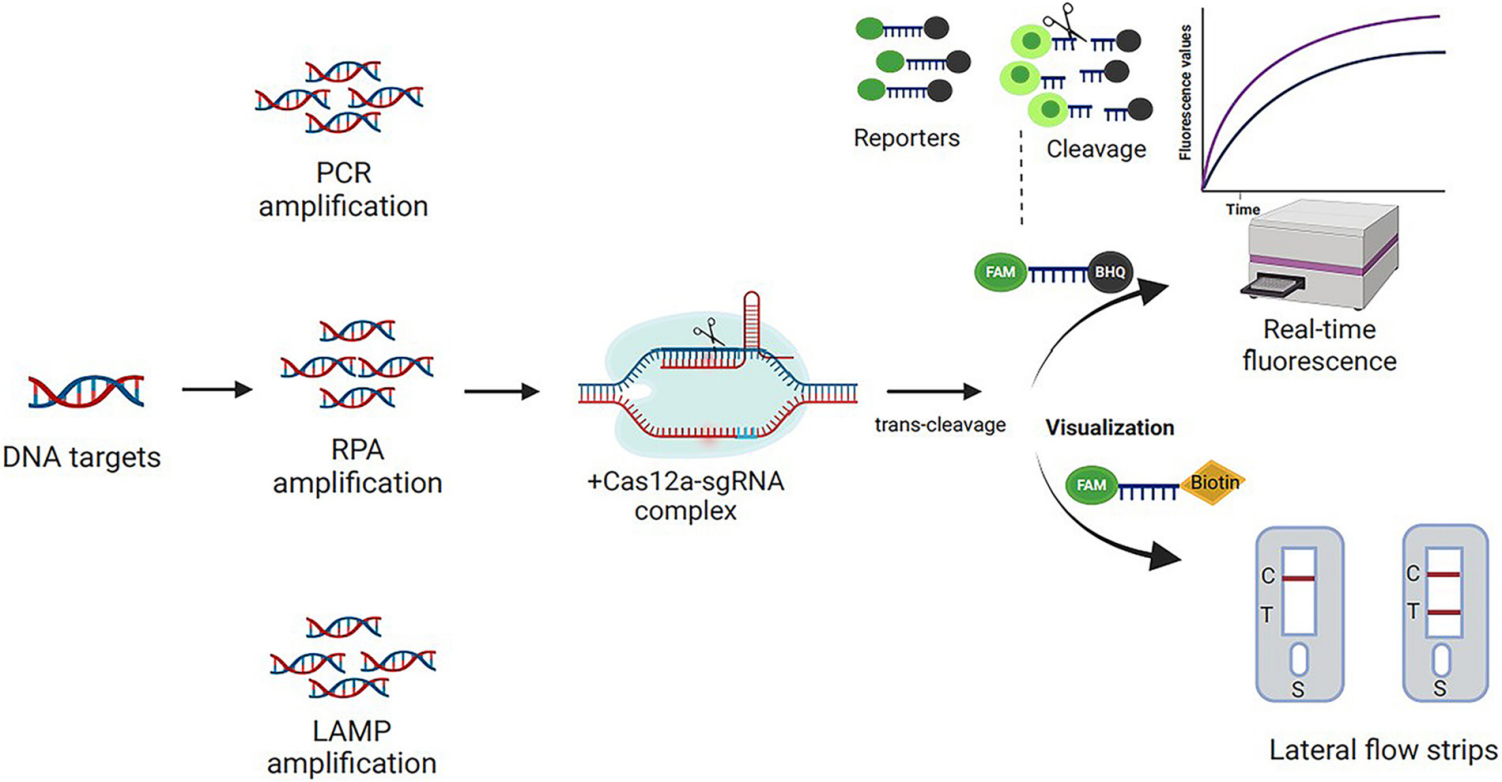
Schematic diagram overview of the study.

Two independent groups recently developed innovative DNA detection platforms based on Cas12a *trans*-cleavage activity: DETECTR and HOLMES (25, 27). Both platforms could accurately detect target DNA by designing specific gRNAs. Consequently, we speculated that a Cas12a-based nucleic acid detection assay could be developed to identify S. aureus and MRSA. Due to its strong discriminatory power in identifying S. aureus strains, the *nuc* gene was chosen as the candidate gene for S. aureus detection (28), and the *mecA* and *nuc* genes were utilized as target genes for MRSA detection (29, 30). In this study, specific sgRNAs targeting the *nuc* and *mecA* genes were designed. As shown in Fig. 1, we first applied PCR, RPA, and LAMP to generate more target DNA templates. The resultant amplicons were subsequently combined with the Cas12a-sgRNA complex. The quenched fluorescent single-stranded-DNA (ssDNA) reporter was *trans*-cleaved upon formation of the Cas12a-sgRNA-target DNA ternary complex, triggering a fluorescence signal that can be monitored using a plate reader. Alternatively, the readout result can also be visualized with lateral-flow strips with a 6-carboxyfluorescein (FAM)-biotin-labeled ssDNA reporter.

### Limit of detection (LOD) of the CRISPR-Cas12a platform.

To validate the activity of sgRNA and develop the CRISPR-Cas12a-based detection platform, recombinant pET-28a(+) plasmids harboring either *nuc* or *mecA* were used for Cas12a reactions.

**FIG 2:**
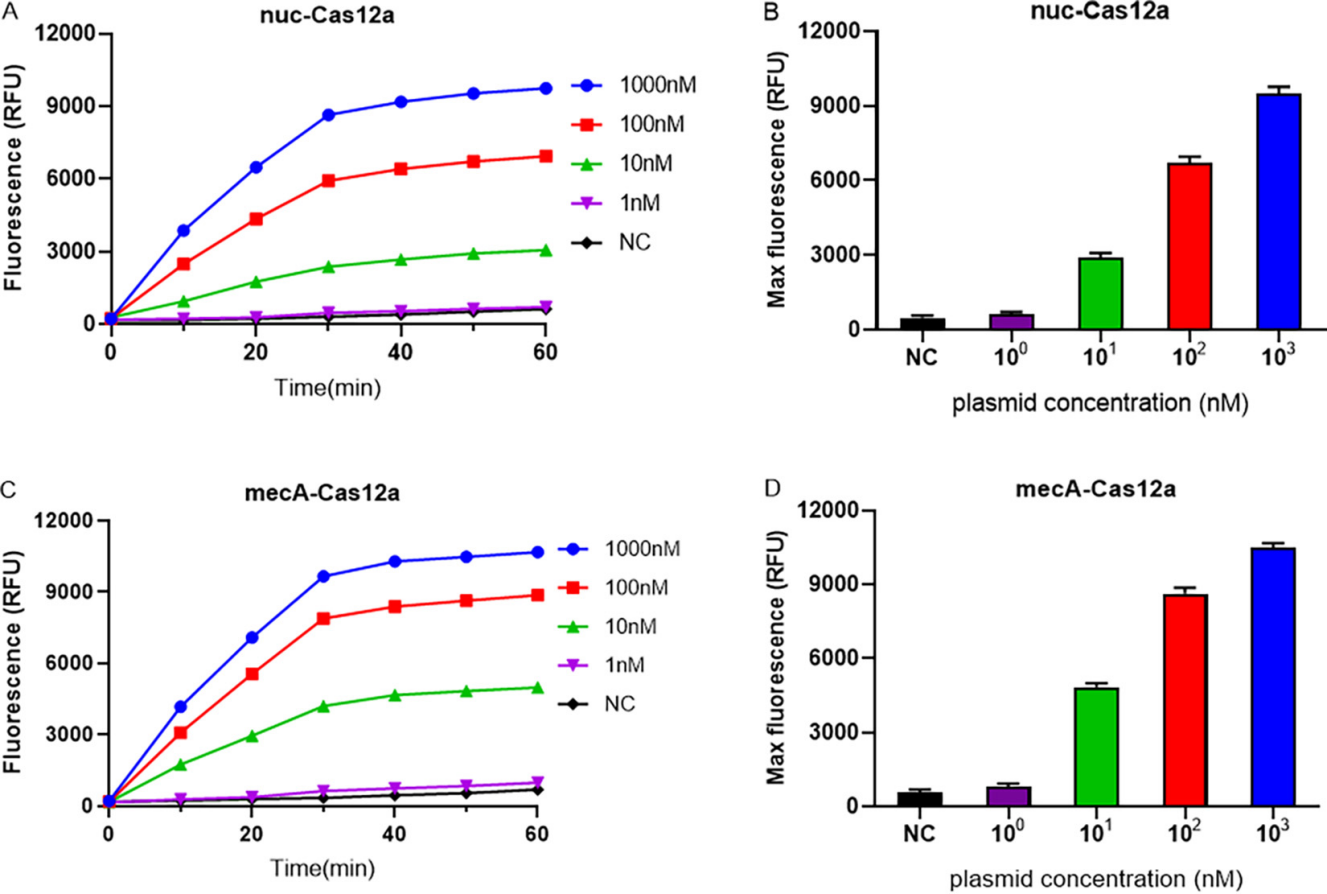
Cas12a detection reactions for the serially diluted plasmids ranging from 1 nM to 1,000 nM. (A and C) Real-time fluorescence signals from 0 to 60 min with different concentration of target plasmids. (B and D) Endpoint fluorescence signals with different concentrations of target plasmids. The negative control (NC) used RNase-free water as input instead of target plasmid. RFU, relative fluorescence units.

Initially, the sensitivity of the CRISPR-Cas12a system was determined using serially diluted plasmids (1 to 1,000 nM) harboring the *nuc* and *mecA* genes, with no nucleic acid amplification steps. As shown in Fig. 2, the fluorescence intensity of the Cas12a reaction system strengthened gradually with increasing reaction duration and DNA concentration, reaching a near maximum at 30 min. In addition, we determined that the CRISPR-Cas12a detection platform has a LOD of 10 nM.

### LOD of the PCR-, LAMP-, and RPA-combined CRISPR-Cas12 platforms.

**FIG 3:**
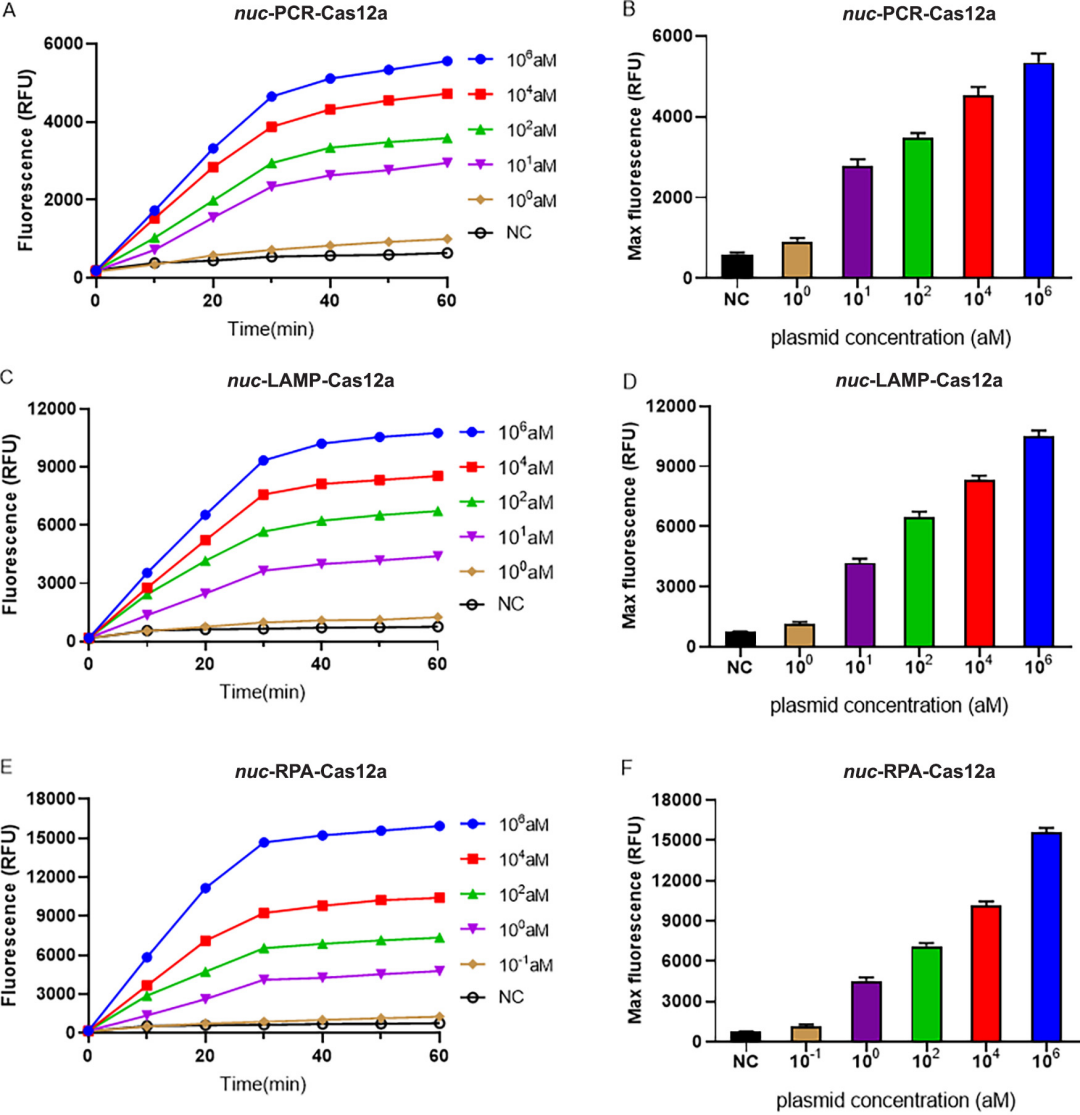
LOD of PCR, LAMP, and RPA combined with the CRISPR-Cas12 platform for the *nuc*-harboring plasmid. (A, C, and E) Real-time fluorescence signals from 0 to 60 min with different concentrations of the target plasmid. (B, D, and F) Endpoint fluorescence signals with different concentrations of the target plasmid. The negative control (NC) used RNase-free water as input instead of target plasmid. RFU, relative fluorescence units.

**FIG 4:**
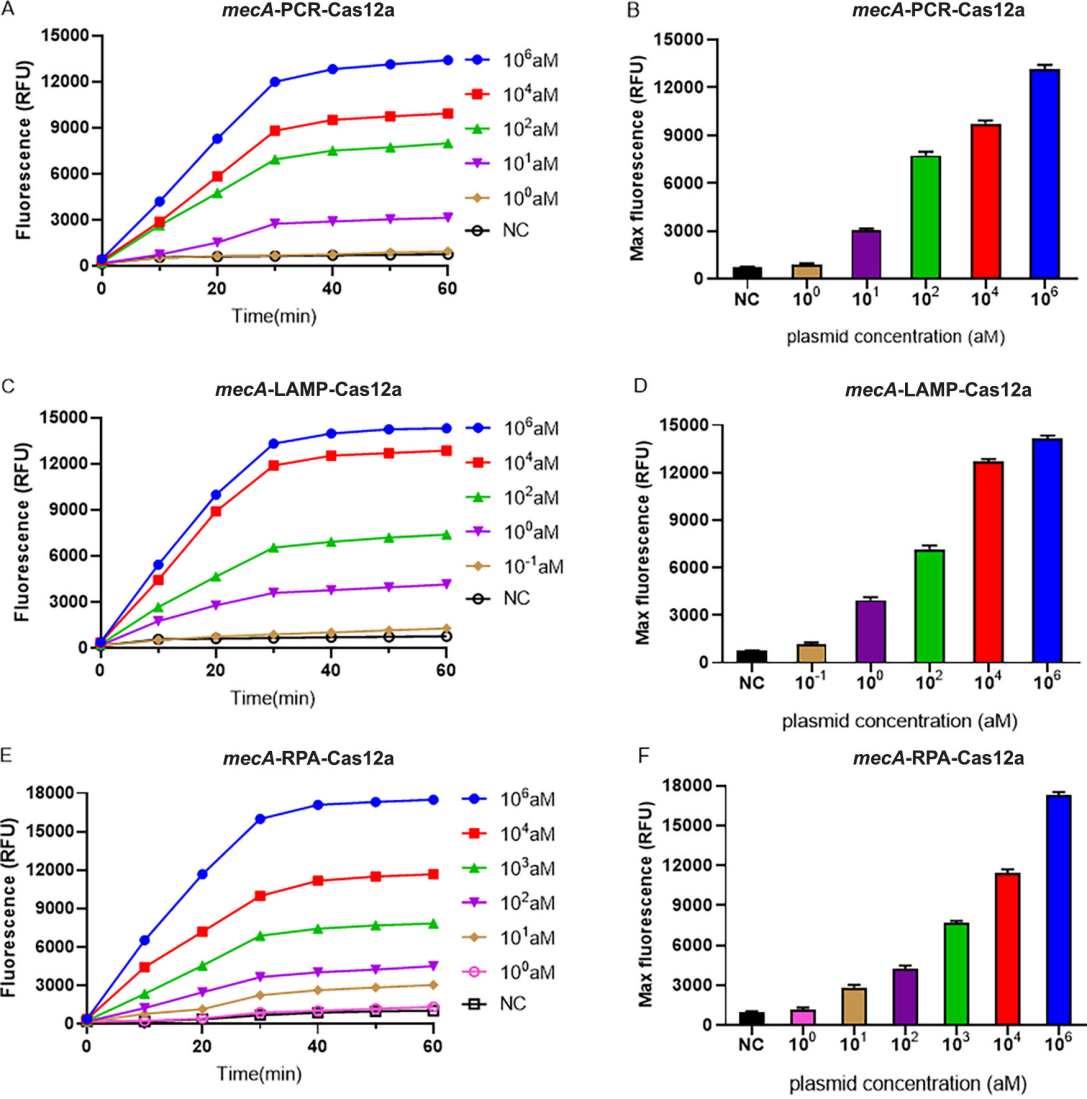
LOD of PCR, LAMP, and RPA combined with the CRISPR-Cas12 platform for the *mecA*-harboring plasmid. (A, C, and E) Real-time fluorescence signals from 0 to 60 min with different concentrations of the target plasmid. (B, D, and F) Endpoint fluorescence signals with different concentrations of the target plasmid. The negative control (NC) used RNase-free water as input instead of the target plasmid. RFU, relative fluorescence units.

In order to increase the sensitivity of the proposed assay, we combined PCR, LAMP, and RPA techniques with the CRISPR-Cas12a system. The LOD of amplification-paired CRISPR-Cas12a systems for the *nuc*- and *mecA*-harboring plasmid were determined. The LOD for *nuc*-PCR-Cas12a-based, *nuc*-LAMP-Cas12a-based, and *nuc*-RPA-Cas12a-based techniques were 10, 10, and 1 aM, respectively (Fig. 3), whereas those for the *mecA* gene-harboring plasmids were 10, 1, and 10 aM, respectively (Fig. 4). Taken together, these results suggested that the *nuc*-RPA-Cas12a-based method for *nuc* gene-harboring plasmids possessed sensitivity superior to that of the *nuc*-PCR-Cas12a-based and *nuc*-LAMP-Cas12a-based methods. In contrast, the *mecA*-LAMP-Cas12a-based detection method for *mecA* gene-harboring plasmids outperformed the other two methods.

### Evaluation of specificity.

**FIG 5:**
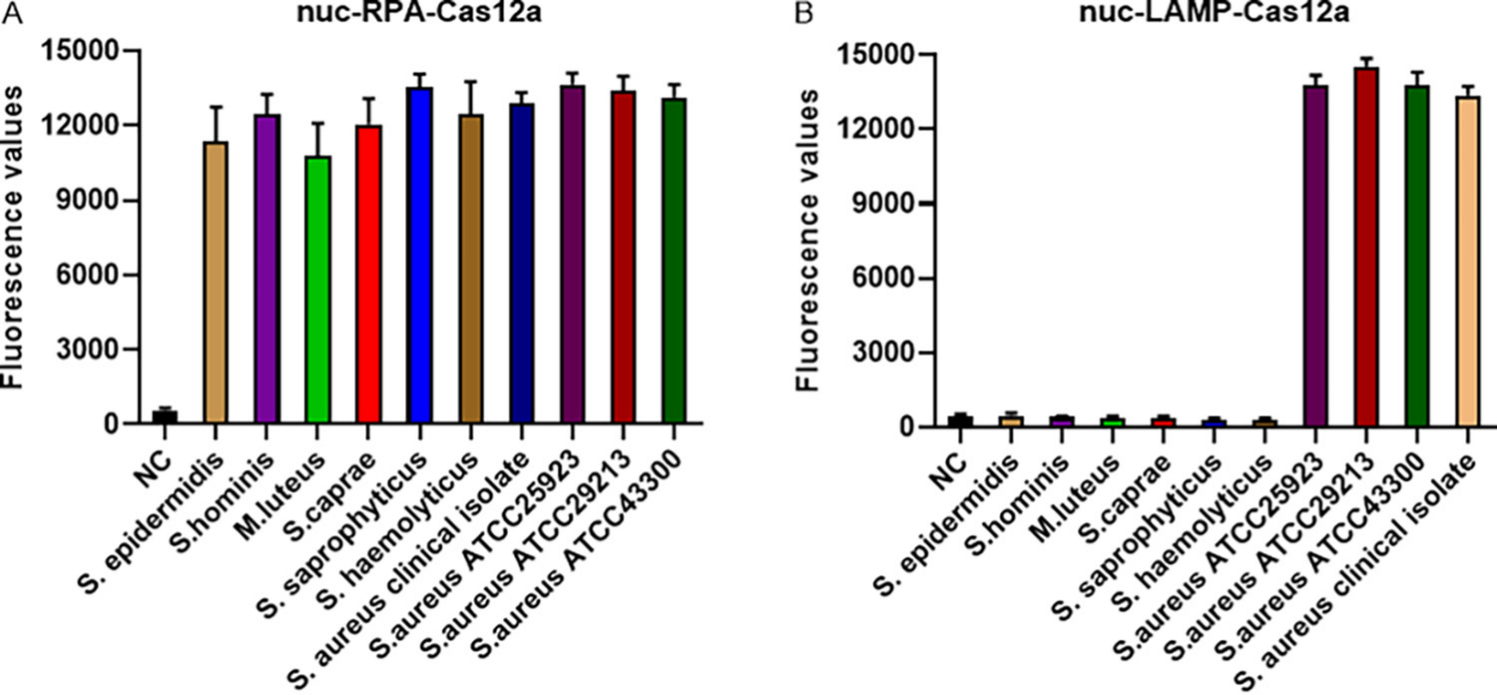
Specificities of the *nuc*-RPA-Cas12a-based (A) and *nuc*-LAMP-Cas12a-based (B) detection methods for the *nuc* gene. The negative control (NC) used RNase-free water as input instead of target plasmid.

**FIG 6:**
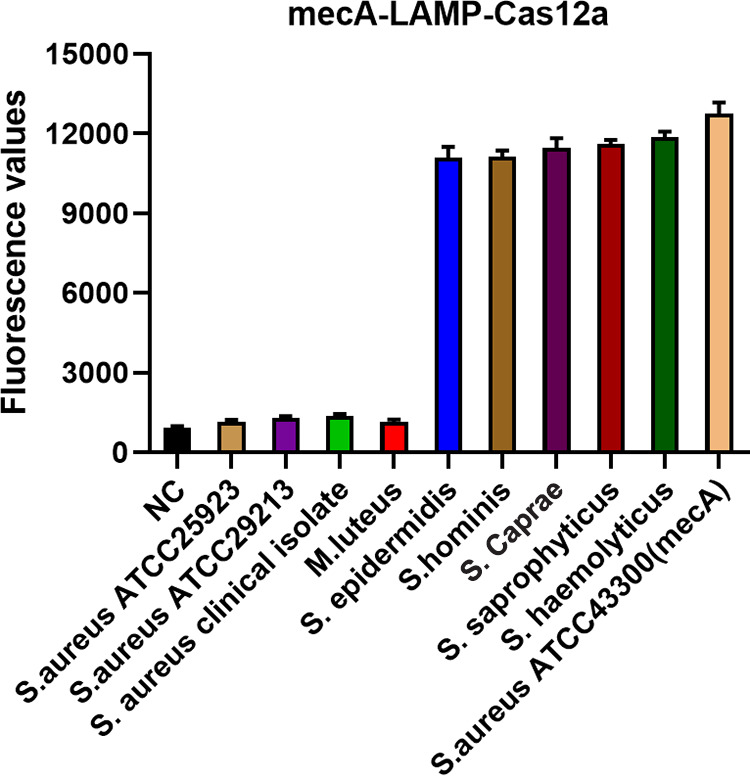
Specificity of the *mecA*-LAMP-Cas12a-based detection methods for the *mecA* gene. The negative control (NC) used RNase-free water as input instead of the target plasmid.

To identify the ideal *nuc* and *mecA* gene detection method, we further evaluated the cross-reactivity of the various methods. The cross-reactivities of *nuc*-RPA-Cas12a-based and *nuc*-LAMP-Cas12a-based methods were determined by evaluating their reactivities to six other bacteria similar to S. aureus (S. epidermidis, S. hominis, Micrococcus luteus, S. caprae, S. saprophyticus, and S. haemolyticus). As illustrated in Fig. 5, the *nuc*-RPA-Cas12a-based method displayed cross-reactivity with the tested bacteria, whereas no cross-reactivity was observed with the *nuc*-LAMP-Cas12a-based method. Concerning the cross-reactivity of the *mecA*-LAMP-Cas12a-based method, its reactivity was evaluated for Staphylococcus strains proven to carry the *mecA* gene (S. epidermidis, S. hominis, *S. caprae*, *S. saprophyticus*, *S. haemolyticus* and S. aureus ATCC 43300) and for *mecA*-negative strains (S. aureus ATCC 25923, S. aureus ATCC 29213, an S. aureus clinical isolate, and M. luteus). As shown in Fig. 6, the *mecA*-LAMP-Cas12a-based method exhibited high specificity with no cross-reactivity.

### Sensitivity of the *nuc*-LAMP-Cas12a and *mecA*-LAMP-Cas12a platforms for MRSA identification.

**FIG 7:**
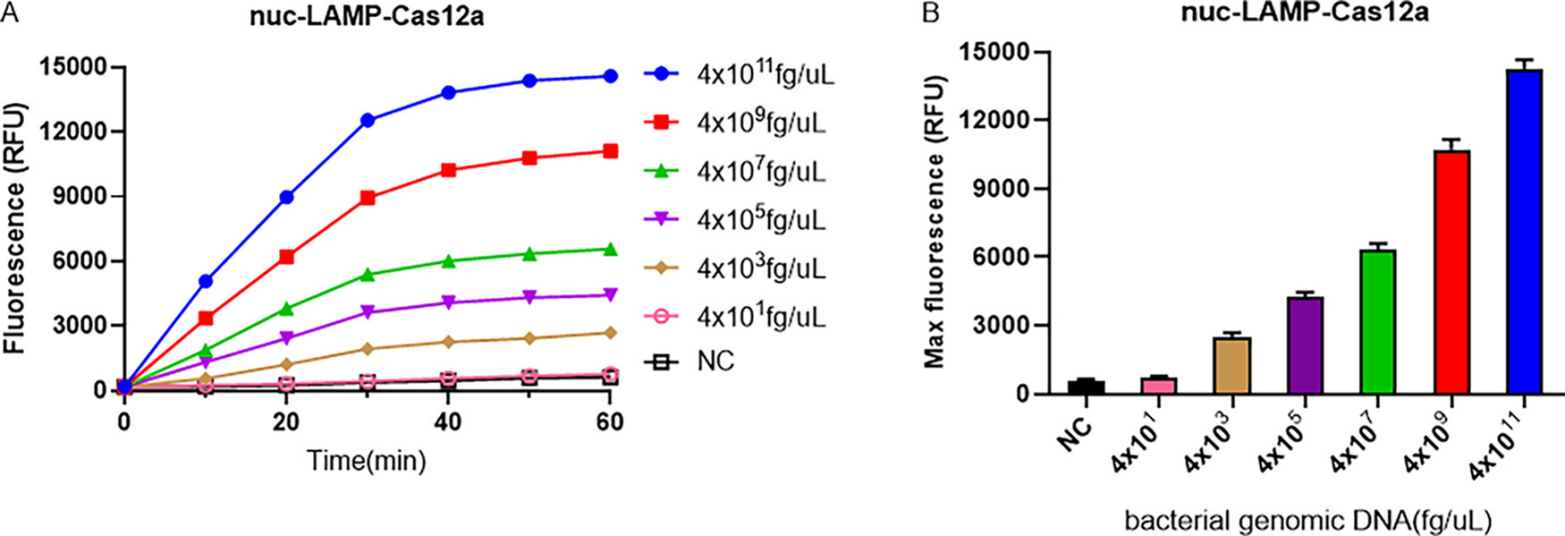
LOD of the *nuc*-LAMP-Cas12a platform for the bacterial genomic DNA of the MRSA isolate. The negative control (NC) used RNase-free water as input instead of the target plasmid. RFU, relative fluorescence units.

**FIG 8:**
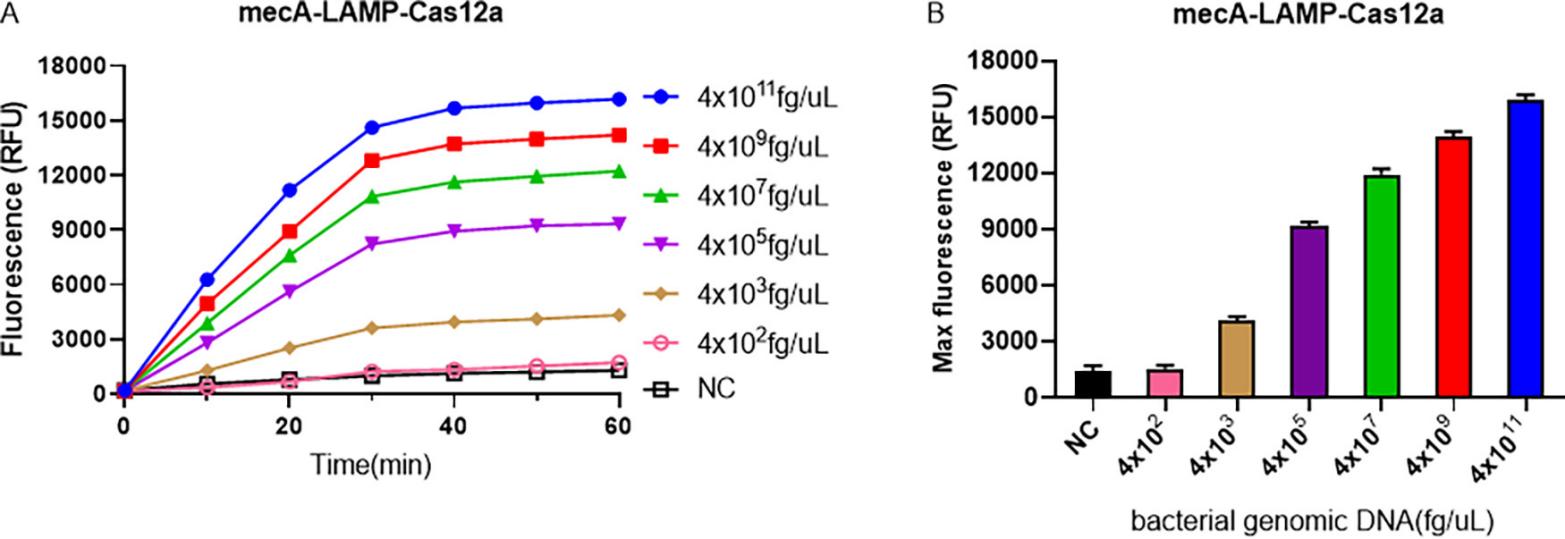
LOD of the *mecA*-LAMP-Cas12a platform for the bacterial genomic DNA of the MRSA isolate. The negative control (NC) used RNase-free water as input instead of target plasmid. RFU, relative fluorescence units.

Based on the above analysis, the best detection methods for *nuc* and *mecA* were the *nuc*-LAMP-Cas12a-based and *mecA*-LAMP-Cas12a-based methods, respectively. To optimize our detection platforms, their performance against bacterial strains was further evaluated. After extraction of the genomic DNA of the MRSA strains, the *nuc*-LAMP-Cas12a and *mecA*-LAMP-Cas12a platforms were used separately to detect serially diluted MRSA bacterial genomic DNA. As shown in Fig. 7 and 8, the LODs of the *nuc*-LAMP-Cas12a and *mecA*-LAMP-Cas12a methods for MRSA identification were both 4 × 10^3^ fg/μL of bacterial genomic DNA.

### Validation of *nuc*-LAMP-Cas12a and *mecA*-LAMP-Cas12a platforms for clinical isolates.

**FIG 9:**
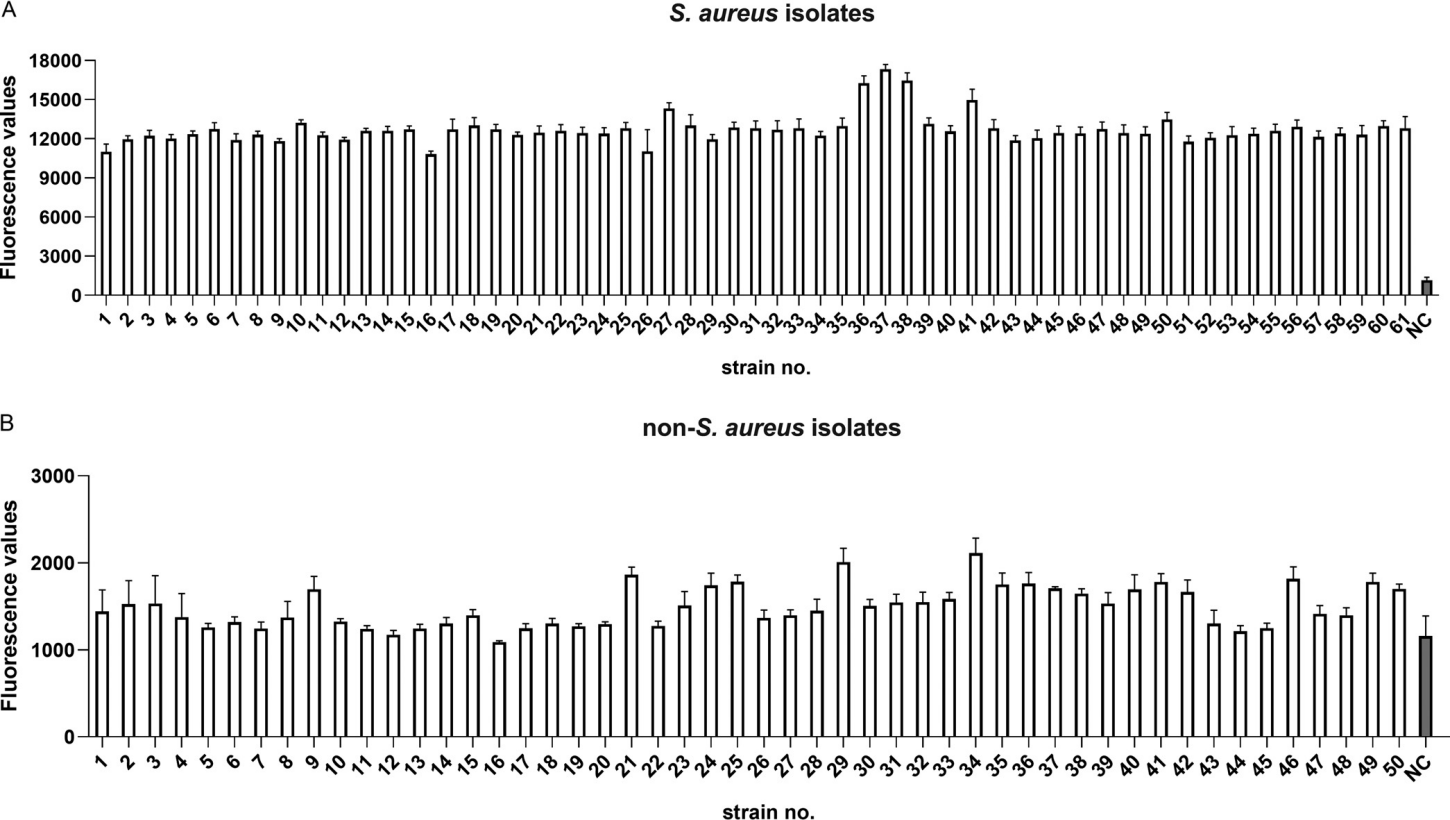
Application of the *nuc*-LAMP-Cas12a platform for clinical S. aureus isolates. The negative control (NC) used RNase-free water as input instead of the target plasmid.

**FIG 10:**
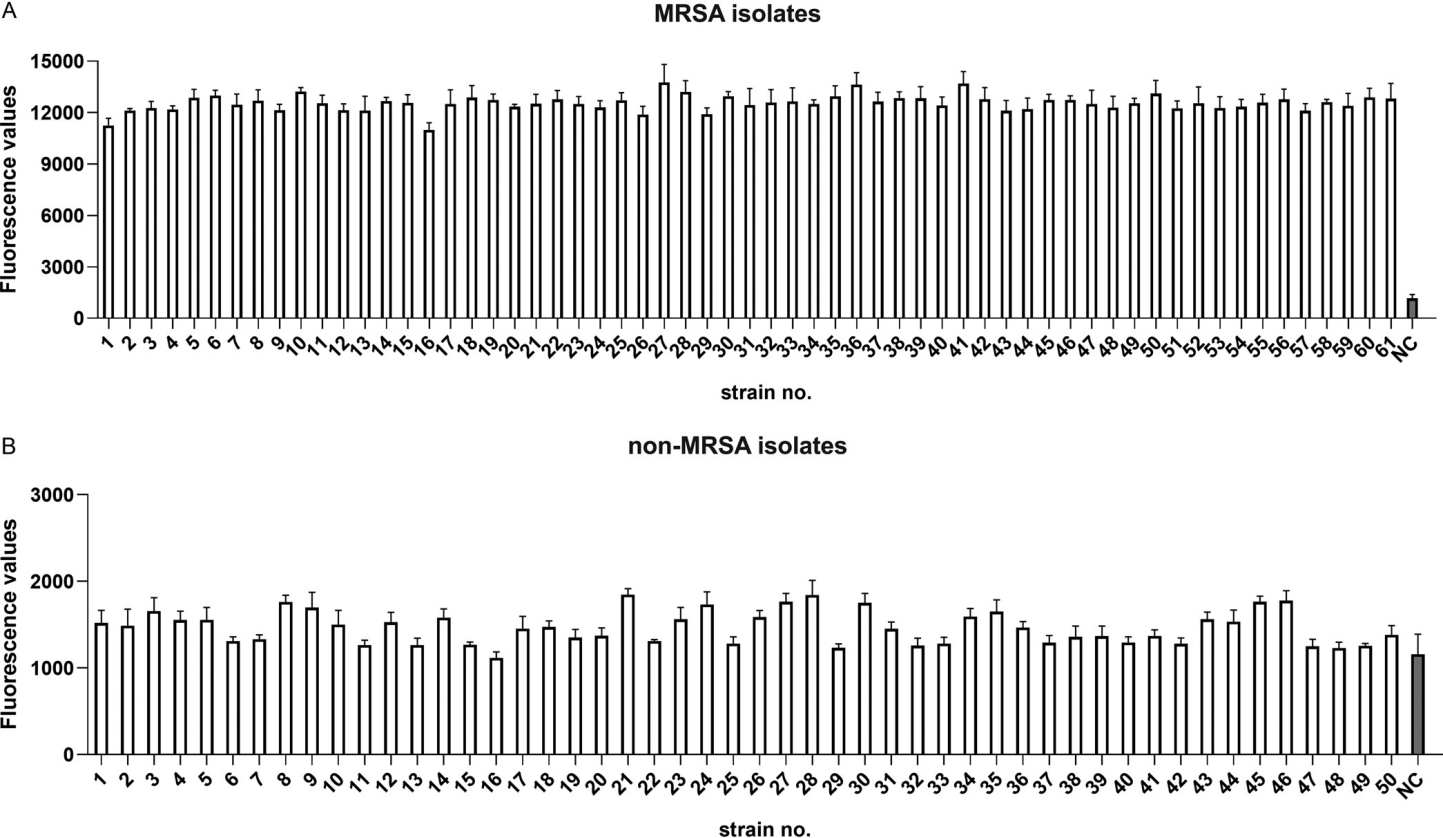
Application of the *mecA*-LAMP-Cas12a platform for clinical MRSA isolates. The negative control (NC) used RNase-free water as input instead of the target plasmid.

To further evaluate the clinical performance of our platforms, 61 MRSA and 50 non-S. aureus clinical isolates were employed. As shown in Fig. 9, the *nuc*-LAMP-Cas12a platform correctly recognized 61 clinical S. aureus isolates while yielding negative results for 50 non-S. aureus isolates. Likewise, with the *mecA*-LAMP-Cas12a detection platform, the *mecA* gene in all 61 clinical MRSA isolates was accurately detected, while the non-MRSA clinical isolates were negative (Fig. 10).

### Validation of the lateral-flow strip visualization system for clinical isolates.

**FIG 11:**
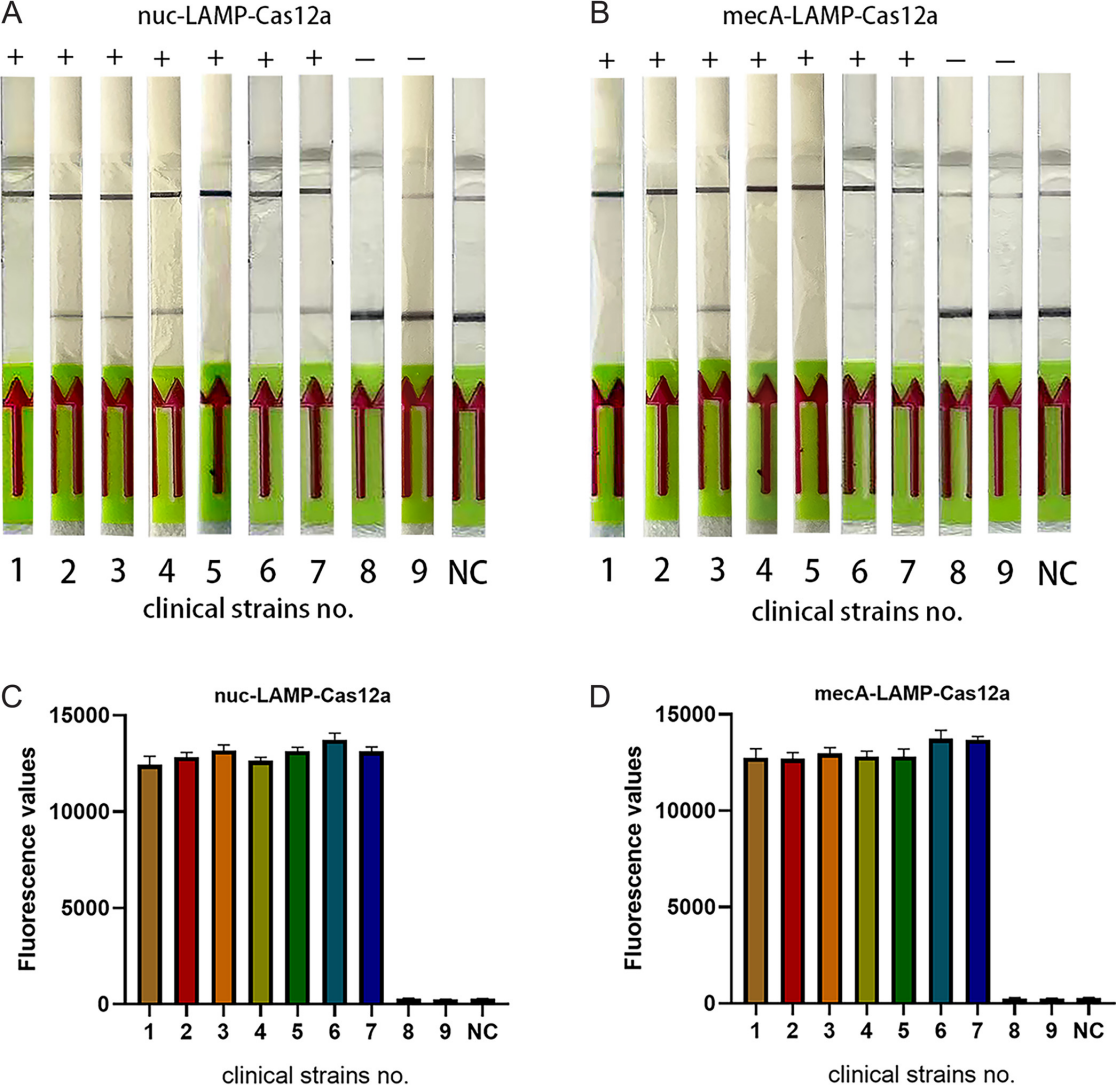
Application of *nuc*-LAMP-Cas12a (A) and *mecA*-LAMP-Cas12a (B) LFS detection assays in clinical isolates. We randomly selected 7 out of 61 clinical MRSA isolates and 2 out of 50 clinical non-*S. aureus* isolates for visual LFS detection. Strains No. 1 to 7 are clinical MRSA isolates, and strains No. 8 and 9 are clinical non-*S. aureus* isolates. C, D respectively represents the endpoint fluorescence result corresponding to A and B LFS results. The negative control (NC) used RNase-free water as input instead of the target plasmid.

Seven strains were randomly chosen from the 61 clinical MRSA isolates, and two strains were randomly selected from the 50 clinical non-MRSA isolates for the visual lateral-flow strip (LFS) detection of the *nuc*-LAMP-Cas12a and *mecA*-LAMP-Cas12a platforms. As depicted in Fig. 11, seven clinical S. aureus strains were accurately recognized, whereas two clinical non-S. aureus isolates were negative for both fluorescence and LFS readout of the *nuc* gene. Similarly, seven clinical MRSA isolates exhibited positive signals for the *mecA* gene, and as expected, the two non-MRSA clinical strains were negative for both fluorescence and LFS readout.

## DISCUSSION

As mentioned above, S. aureus is spreading globally and poses a growing health risk (31). Specifically, the genetic adaptability of MRSA and the recurrent emergence of successful epidemic strains make it a major threat to human health (32). The global MRSA pandemic underscores the need for diagnostic tests that are rapid, accurate, cost-effective, and deployable at the point of care, which would aid in both infection control and patient management. Conventional bacterial culture, RT-PCR, mass spectrometry, and metagenomic next-generation sequencing (mNGS) are current techniques for S. aureus and MRSA detections, which all have long turnaround times, require well-trained personnel, and rely on expensive equipment (33–35). These drawbacks are accentuated in limited-resource settings and at the point of care. Isothermal amplification techniques, such as LAMP and RPA, do not require specific instrumentation and qualified personnel; nevertheless, they have the disadvantages of nonspecific binding and false-positivity issues. By combining the isothermal amplification technique with the CRISPR-Cas12a system, nucleic acid can sensitively and specifically be detected.

This study set out with the aim of developing a fast and accurate S. aureus and MRSA detection platform by combining the CRISPR-Cas12a system with the PCR, LAMP, and RPA techniques. The *nuc*-LAMP-Cas12a and *mecA*-LAMP-Cas12a platforms were demonstrated to be able to detect S. aureus and MRSA with 100% sensitivity and 100% specificity in a total of 111 clinical isolates. On the other hand, from the commencement of template preparation to signal detection, the *nuc*-LAMP-Cas12a and *mecA*-LAMP-Cas12a detection assays took only 80 min (with fluorescence readout) or 85 min (with strip readout). Therefore, they were deemed promising tools for rapid and accurate identification of S. aureus and MRSA. The benefit of these rapid platforms would be even greater outside clinical laboratory settings where diagnostic equipment is inaccessible or where a speedy turnaround is required.

By designing specific sgRNAs targeting the *nuc* and *mecA* genes, we initially developed CRISPR-Cas12a detection systems employing real-time and endpoint fluorescence for signal detection. Without nucleic acid amplification, they were able to detect plasmids individually harboring the *nuc* and *mecA* genes at concentrations as low as 10 nM, which is on par with the findings of earlier investigations in which the LOD for the CRISPR-Cas12a system was between 10 and 100 nM for different targets (12, 36). In order to further close the gap in sensitivity, we combined PCR, LAMP, and RPA techniques into the CRISPR-Cas12a system. In terms of sensitivity, the *nuc*-RPA-Cas12a-based method was the most sensitive method for *nuc* gene detection, while the *mecA*-LAMP-Cas12a-based method demonstrated superior sensitivity for *mecA* identification. Concerning cross-reactivity, the *nuc*-RPA-Cas12a-based method exhibited cross-reactivity with the tested bacteria, whereas the *nuc*-LAMP-Cas12a-based method did not. Based on these results, the *nuc*-LAMP-Cas12a-based method was deemed the optimal detection method for S. aureus detection. On the other hand, as the *mecA*-LAMP-Cas12a-based method demonstrated high specificity without any cross-reactivity, it was selected as the optimal method for *mecA* gene identification. Both the *nuc*-LAMP-Cas12a-based and *mecA*-LAMP-Cas12a-based methods showed LOD of 4 × 10^3^ fg/μL of bacterial genomic DNA, which was as sensitive as those of earlier investigations in which the LOD was attomolar levels (26). For further evaluation of the clinical performance of our platforms, we critically validated the *nuc*-LAMP-Cas12a-based and *mecA*-LAMP-Cas12a-based methods on 111 clinical bacterial isolates and found them to be 100% specific and 100% sensitive with both the fluorescence readout and the lateral-flow readout (Fig. 9 and 10), demonstrating that these two platforms could sensitively and specifically identify S. aureus and MRSA isolates. Therefore, the *nuc*-LAMP-Cas12a-based and *mecA*-LAMP-Cas12a-based methods were selected as the optimal platforms for *nuc* and *mecA* detection, respectively.

One advantage of the present platforms was the short turnaround time. The entire detection procedure included template and reagent preparation, LAMP, and Cas12a reaction. The time required for nucleic acid extraction can vary significantly depending upon the DNA extraction technique used. In this study, an approach involving liquid nitrogen freezing and crushing was employed, which took about 30 min. Previous research indicated that LAMP took at least 15 min (26, 37); according to our assays, 20 min was sufficient for LAMP. For the optimal Cas12a reaction time, the real-time fluorescence readout was used to monitor the entire Cas12a reaction response. Results in this study suggested that while 30 min was the optimal time for Cas12a *trans*-cleavage signal detection, 5 min was the optimal time for lateral-flow strip imaging (38). That is to say, the *nuc*-LAMP-Cas12a and *mecA*-LAMP-Cas12a detection assays took around 80 min (based on fluorescence readout) or 85 min (based on strip readout). Another advantage of the present platforms is that the results can be interpreted by the naked eye. Our proposed methods used a simple readout system akin to pregnancy tests, with results displayed on a paper strip and visible to the naked eye. Thus, this study provided a broadly applicable, rapid, simple, and accurate nucleic acid platform for S. aureus and MRSA detection. This is crucial in limited-resource areas, as expensive equipment for molecular experiments might not be available in low- and middle-income countries.

Despite the fact that the developed platforms showed 100% positive and negative predictive values, due to the limited number of clinical strains investigated, the results should still be interpreted with caution, and a large, multicenter cohort study utilizing more clinical isolates is needed to further confirm the clinical performance of our assays. Also, clinical validation of the present assay for the detection of S. aureus and MRSA from original clinical samples is needed. The three-step protocol evaluated here is troublesome to operate outside the molecular diagnostic laboratory. By combining genetic amplification and CRISPR-based detection into one step, a one-pot CRISPR diagnostic protocol for the multiplexed detection of *nuc* and *mecA* will be much easier to perform. Beyond S. aureus and MRSA, we hope that similar CRISPR diagnostic platforms will have long-lasting application in the detection of various pathogens, cancers, pharmacogenetics, food contamination, and gene mutations.

### Conclusion.

In conclusion, fast and accurate S. aureus and MRSA detection platforms with 100% sensitivity and specificity were successfully established by combining isothermal amplification and a CRISPR-Cas12a system. The *nuc*-LAMP-Cas12a and *mecA*-LAMP-Cas12a platforms were the optimal *nuc* and *mecA* gene detection methods, respectively. A key strength of the present work was the use of thermostatic amplification technology in conjunction with lateral-flow test strip technology without using sophisticated molecular experimental equipment, thus allowing for point-of-care testing. Future work can further improve the user-friendliness of the present assay by creating a one-pot protocol for the multiplex detection of *nuc* and *mecA*.

## MATERIALS AND METHODS

### Design and synthesis of nucleic acid targets and sgRNAs.

Due to its strong discriminatory power in identifying S. aureus strains, the thermonuclease (*nuc*) gene was chosen as the candidate gene for S. aureus detection (28), and the *mecA* and *nuc* genes were utilized as target genes for MRSA identification. The *nuc* and *mecA* gene sequences of MRSA strains were downloaded from NCBI (https://www.ncbi.nlm.nih.gov/). Using MEGA 7 software, several *nuc* and *mecA* gene sequence alignments were carried out. Following sequence alignment, corresponding specific sgRNAs were designed based on the conserved sequences of the *nuc* and *mecA* genes that matched the Cas12a protospacer adjacent motif (5′-TTTN) (see Table S1 in the supplemental material). sgRNAs were subsequently synthesized by TaKaRa Bio (Beijing, China). Using the Basic Local Alignment Search Tool (BLAST [http://blast.ncbi.nlm.nih.gov/Blast.cgi]), the specificity of the candidate target sequences of the *nuc* and *mecA* genes was determined, as was sequence alignment. In addition, partial sequences of the *nuc* and *mecA* genes were synthesized by Sangon Biotech (Shanghai, China), cloned into pET-28a vectors, and transformed into TOP10 competent cells. Recombinant pET-28a vectors harboring the *nuc* or *mecA* gene were extracted using a miniprep plasmid extraction kit (Qiagen, Hilden, Germany).

### Cas12a detection reactions.

The Cas12a *trans*-cleavage experiment was performed with EnGen LbaCas12a (New England Biolabs [NEB], Ipswich, MA, USA). Initially, 1 μM LbaCas12a was preassembled for 10 min at room temperature with 1.25 μM sgRNA (TaKaRa Bio, Beijing, China) and 10 U RNase inhibitor (TaKaRa Bio). The DNA target was then dissolved in 10× NE buffer 2.1 (NEB) solution and mixed with LbCas12a-sgRNA complexes, 500 nM custom ssDNA reporter (Sangon Biotech, Chengdu, China), and double-distilled water (ddH_2_O) to a final volume of 20 μL. The final concentrations of LbaCas12a and sgRNA in the solution were 50 and 62.5 nM, respectively. Finally, the 20-μL reaction mixtures were transferred to a 384-well microplate (Corning Life Sciences, Corning, NY, USA), and real-time and endpoint fluorescence signals were acquired using a fluorescence plate reader (Tecan Infinite 200 Pro; Tecan, Grödig, Austria) every 60 s for up to 60 min at 37°C (ssDNA Fluorophore-quencher (FQ) reporter: λ_ex_, 492 nm; λ_em_, 522 nm). The Cas12a *trans*-cleavage signals were also identified using lateral-flow strips (Milenia Hybridetect 1; TwistDx, Cambridge, UK) in accordance with the manufacturer’s instructions, using an ssDNA Fluorophore-biotin (FB) reporter labeled with 6-FAM and biotin at both ends.

### PCR and Cas12a detection reaction.

Based on the target sequences of the sgRNAs for the *nuc* and *mecA* genes, the PCR primers were designed using the Primer3Plus (http://primer3plus.com/) software. PCR primer specificity analysis and sequence alignment were conducted using BLAST (http://blast.ncbi.nlm.nih.gov/Blast.cgi). The *nuc* and *mecA* genes were amplified by PCR using the aforementioned primer pairs. The PCR products were then examined using 1% agarose gel electrophoresis and sequenced directly. Using 1 μL of unpurified PCR products as DNA targets, we then performed the Cas12a detection assay as described above.

### LAMP and Cas12a detection reaction.

Based on the target sequences of the sgRNAs for the *nuc* and *mecA* genes, LAMP primers were designed using the PrimerExplorer v5 software (http://primerexplorer.jp/lampv5e/index.html). BLAST was used for both LAMP primer specificity analysis and sequence alignment. The LAMP procedure was conducted using a WarmStart colorimetric LAMP 2× master mix kit (DNA and RNA; New England Biolabs, Hitchin, United Kingdom) according to the manufacturer’s instructions. Following this, 1 μL of unpurified LAMP amplified product was used as target DNA to conduct the Cas12a detection test as described above.

### RPA and Cas12a detection reaction.

Based on the target sequences of the sgRNAs for the *nuc* and *mecA* genes, RPA primers were designed using the Primer3Plus software following the manufacturer's instructions (TwistDx). Both RPA primer specificity analysis and sequence alignment were conducted using BLAST. The RPA procedure was conducted using a TwistAmp Basic kit (TwistDx) according to the manufacturer’s instructions. Following this, 1 μL of unpurified RPA amplified product was used to supplement the Cas12a detection assay as described above.

### Bacterial strain collection and identification.

In the present study, the reference strains, including S. aureus ATCC 43300, S. aureus ATCC 25923, and S. aureus ATCC 29213, were provided by the Department of Clinical Laboratory of the First Affiliated Hospital of Chongqing Medical University. In addition, all the remaining bacterial strains were isolated from clinical samples by the microbiology laboratory of the Department of Clinical Laboratory of the First Affiliated Hospital of Chongqing Medical University (Table S2). The genomic DNA of bacterial strains was extracted using a bacterial genomic DNA extraction kit (Tiangen, Beijing, China) according to the manufacturer’s instructions. All clinical isolates were identified using a Vitek 2 Compact system (bioMérieux, Inc., Durham, NC, USA). MRSA was identified through PCR amplification and Sanger sequencing.

### Evaluation of sensitivity and specificity.

Using serially diluted plasmids (1 to 1,000 nM) harboring the *nuc* and *mecA* genes, the sensitivity of the CRISPR-Cas12a system was first evaluated without any nucleic acid amplification steps. In order to boost its sensitivity, we incorporated PCR, LAMP, and RPA methods separately into the CRISPR-Cas12a system. To determine the ideal strategy for identifying the *nuc* and *mecA* genes, we compared the sensitivity and specificity of the three different amplification methods combined with the CRISPR-Cas12a platform. The sensitivity of the three platforms was evaluated using serially diluted plasmids (1 to 10^6^ aM) carrying *nuc* and *mecA* genes. In addition, four S. aureus strains and six other similar bacterial strains (Staphylococcus epidermidis, Staphylococcus hominis, Micrococcus luteus, Staphylococcus caprae, Staphylococcus saprophyticus, and Staphylococcus haemolyticus) were used to evaluate the specificity of the three platforms for the *nuc* gene. Staphylococcus species strains harboring the *mecA* gene (S. epidermidis, S. hominis, *S. caprae*, *S. saprophyticus*, *S. haemolyticus*, *and* S. aureus ATCC 43300), and *mecA*-negative bacterial strains (S. aureus ATCC 25923, S. aureus ATCC 29213, S. aureus clinical isolate, and M. luteus) were used to evaluate the specificity of the three platforms for the *mecA* gene. Using serially diluted MRSA bacterial genomic DNA (4 × 10^0^ to 4 × 10^11^ fg/μL), we further assessed the sensitivity of our methods for the MRSA strain. Finally, the clinical performance of our methods for *nuc* and *mecA* gene identification was validated by analyzing 61 clinical MRSA isolates and 50 other commonly isolated clinical strains, including six Enterococcus faecalis, six Enterococcus faecium, six S. epidermidis, six Streptococcus pneumoniae, six Escherichia coli, five Klebsiella pneumoniae, five Acinetobacter baumannii, five Pseudomonas aeruginosa, and five Enterobacter cloacae isolates.

### Statistics.

The Cas12a detection reactions were replicated at least three times in three parallel experiments. In all figures, data are means and standard errors of the means (SEM). Graph construction and statistical analysis were performed using GraphPad Prism v.8.1.2 software (GraphPad Software, San Diego, CA, USA).

### Ethical statement.

The collection of bacterial strains followed the relevant clinical practice guidelines. All patient identifications were removed during the trials; thus, ethical approval was not required for this study.
